# Prognostic Impact of *HOTAIR* Expression is Restricted to ER-Negative Breast Cancers

**DOI:** 10.1038/srep08765

**Published:** 2015-03-05

**Authors:** Yesim Gökmen-Polar, I. Tudor Vladislav, Yaseswini Neelamraju, Sarath C. Janga, Sunil Badve

**Affiliations:** 1 Department of Pathology and Laboratory Medicine, Indiana University School of Medicine, Indianapolis, IN; 2 Department of Medicine, Indiana University School of Medicine, Indianapolis, IN; 3 Department of Biohealth Informatics, School of Informatics and Computing, IUPUI, Indianapolis, IN; 4 Indiana University Melvin and Bren Simon Cancer Center, Indianapolis, IN

## Abstract

Expression of *HOX* transcript antisense intergenic RNA (*HOTAIR*), a large intergenic noncoding RNA (lincRNA), has been described as a metastases-associated lincRNA in various cancers including breast, liver and colon cancer cancers. We sought to determine if expression of *HOTAIR* could be used as a surrogate for assessing nodal metastases and evaluated RNA in situ hybridization (RNA-ISH) assay in a tissue microarray constructed from 133 breast cancer patients. The prognostic value of *HOTAIR* was further validated in large cohorts using The Cancer Genome Atlas (TCGA) breast cancer subjects. RNA-ISH analysis was successful in 94 cases (17% cases scored 0, 32.9% scored 1, 30.8% scored 2, and 19.1% scored 3). The expression of *HOTAIR* did not correlate with nodal metastasis regardless of the scoring intensity or with other study parameters (age, tumor size and grade, expression status). Further analysis of TCGA dataset showed that *HOTAIR* expression was lower in ductal carcinomas but higher in ER-negative tumors. Overexpression of *HOTAIR* was not associated with nodal metastases or prognosis in ER-positive patients. Its function as a poor prognostic indicator in ER-negative patients was restricted to node-positive patients. *HOTAIR* appears to be a marker for lymphatic metastases rather than hematogenous metastases in ER-negative patients.

Recent studies have shown that the idea of DNA transcription resulting in synthesis of corresponding protein is rather simplistic^1^. A number of additional factors are involved in the control of the transcription process. RNAs do not appear to be simple messengers but are complex species with many distinct functional subtypes. Next generation sequencing studies have estimated that 98% of the DNA is noncoding; these RNAs derived from noncoding regions have a variety of functions that include gene regulation^2^,^3^. Among the different classes of noncoding (ncRNAs), microRNAs have been the most extensively investigated^4^. In addition to microRNAs, long noncoding RNAs (lncRNAs) constitute another species of RNAs. LncRNAs are defined as transcribed RNA molecules that are longer than 200 nucleotides and have no obvious protein coding capacity^5^. They are more tissue specific than protein coding genes^6^. The abundance of lncRNAs in the genome, their association with the myriad of different disease phenotypes, and their diverse cellular roles are drawing the attention of the scientific community^7^,^8^,^9^,^10^,^11^,^12^,^13^,^14^,^15^. However, knowledge regarding the actual number of functional lncRNAs and the mechanism(s) by which they carry out their functions is still limited. Recent advances in RNA sequencing (RNA-seq) and computational methods have revealed that large intergenic noncoding RNAs (lincRNAs) are the largest class of lncRNA molecules in the human genome^5^. At least 8000 human lincRNAs have been identified^5^. Biological characterization studies suggest that many lincRNAs act as scaffolds that regulate molecular (protein, RNA, and DNA) interactions required for various signaling networks. It has been estimated that at least 30% of lncRNAs are bound to the polycomb repressive complexes and target these chromatin-modifying complexes to the target genes^16^.

Gupta et al hybridized RNA from normal breast epithelia, primary tumors, and distant metastases to ultrahigh density *HOX* tiling array^17^. They found 170 ncRNAs and 63 HOX exons that were differentially expressed. *HOTAIR* (*HOX* antisense intergenic RNA) was one of the metastases associated lincRNAs. Rinn et al and Woo and Kingston et al had previously identified its role in regulating *HOX* genes^18^,^19^. *HOTAIR* is a large noncoding RNA which is 2158-nucleotides long, and expressed from the *HOXC* locus on chromosome 12q13.13^18^. It contains 5′ and 3′ domains. The 5′ domain binds to PRC2, while the 3′ domain interacts with the LSD1/Co-REST/REST complex to coordinately regulate histone H3 lysine 27 methylation and lysine 4 demethylation, and epigenetically modify chromatin structure thereby regulating gene expression^20^. Gupta et al further showed by qPCR that *HOTAIR* is overexpressed 100–2000 fold in breast cancer metastases^17^. Its expression was sometimes high but heterogeneous in primary tumors. The expression was a significant predictor of metastases and death in a series of 132 breast cancer patients with extensive followup.

Since the original publication, *HOTAIR* has been shown to be a poor prognostic factor in a number of cancers including breast, lung, colon, liver and gastrointestinal stromal tumors^17^,^21^,^22^,^23^,^24^. Its expression has been linked to increased cancer cell invasiveness and epithelial-to-mesenchymal transformation^25^. Gain-of-function studies show that the activities of hundreds of genes (such as the *HOXD* locus, progesterone receptor, cell adhesion molecules) are inhibited, while dozens of other genes (such as *ABL2, SNAIL*, and *LAMB3*) are activated^17^. An in vitro functional study showed that *HOTAIR* overexpression in four different breast cancer cell lines could promote colony growth and invasion^17^. Animal experiments demonstrated that the breast cancer cell line, MDA-MB-231, which expressed *HOTAIR*, grew quickly in primary tumor foci with increased metastatic potential to the lung^17^.

Given the purported importance of *HOTAIR* in breast cancer, we sought to address the question of whether the expression of *HOTAIR* could be used as a surrogate for assessing nodal metastases. We additionally analyzed the impact of high levels of *HOTAIR* on nodal metastases and overall survival in The Cancer Genome Atlas (TCGA) dataset.

## Results

### In situ *HOTAIR* Expression in Breast Cancer TMA Cohort

The expression of *HOTAIR* was analyzed in 94 of the 133 cases by RNA-ISH based on the eligibility criteria. These criteria included the presence of at least 100 tumor cell nuclei. Thirty-nine cases were excluded due to lack of adequate tumor cells, folds in tissue sections, or loss of tissue during processing.

**Figure 1:**
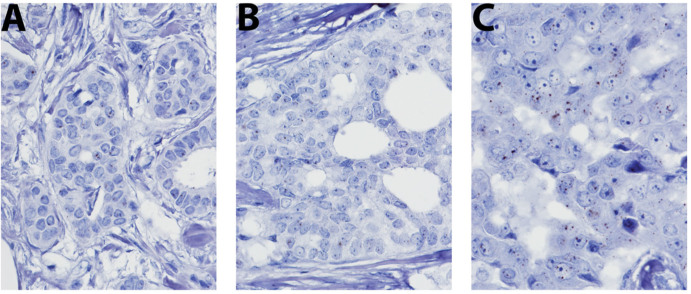
Expression of *HOTAIR* in a series of breast cancer samples. Expression levels were depicted as (A) 1–10 signals, (B) 10–100 signals, and (C) multiple signals per cell.

Consistent with prior descriptions, the signals were scattered in the cell and were not restricted to the nucleus^26^. Similarly, very few, if any, signals were identified in the nontumor stromal cells—highlighting the tissue-specific distribution of this lincRNA. The signals were absent or rare (less than 1 per 100 tumor nuclei) in 16 cases (17%); one to 10 signals per 100 nuclei in 31 cases (32.9%), 10 to 100 signals in 29 (30.8%), and innumerable in 18 cases (19.1%). Representative images of *HOTAIR* microRNA signals are shown in Figure 1A–C for each scoring category. The expression of *HOTAIR* did not correlate with nodal metastasis regardless of the scoring intensity used as a cutoff point (Table 1). It also did not correlate with other parameters such as age (categorical 50 versus > 50), tumor size and grade, and ER and HER2 status.

### Confirmation of the Prognostic Value of *HOTAIR* in Breast Cancer Using TCGA Breast Invasive Carcinoma Dataset

**Figure 2:**
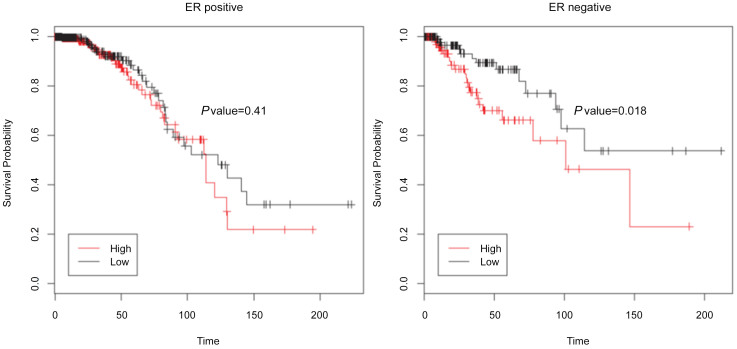
Impact of *HOTAIR* expression on survival in The Cancer Genome Atlas dataset (n = 952). KM plots show that *HOTAIR* expression above median is associated with poor outcome in ER-negative patients (A) but not in ER-positive patients (B).

To assess the prognostic value of *HOTAIR* expression in breast tumors, we next analyzed its correlation with overall survival using TCGA data of breast cancer subjects. The expression of *HOTAIR* in these subjects was categorized based on the low (n = 476; black line) and high (n = 476; red line) expressions using the median expression as a cutoff (Figure 2A–B). The higher *HOTAIR* expression was associated with shorter overall survival in ER-negative breast cancer patients (*P* = 0.018) (Figure 2B). In the cohort with high *HOTAIR* levels, the overall survival probability was 60% and 46.4% at 50 and 100 months, respectively, and those with low *HOTAIR* levels showed a survival probability of 86% and 62.8% at 50 and 100 months, respectively. On the other hand, *HOTAIR* levels were not associated with overall survival in ER-positive breast cancer (*P* = 0.41) (Figure 2A).

**Figure 3:**
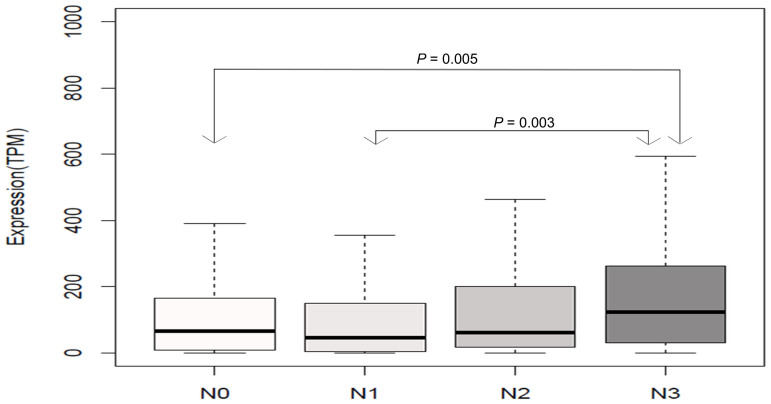
Correlation of *HOTAIR* expression with nodal status using The Cancer Genome Atlas dataset. Comparison of the cases by nodal status revealed only significant association between N3 versus N0 (*P* = 0.005) or N1 (*P* = 0.003). Other comparisons were not significant.

**Figure 4:**
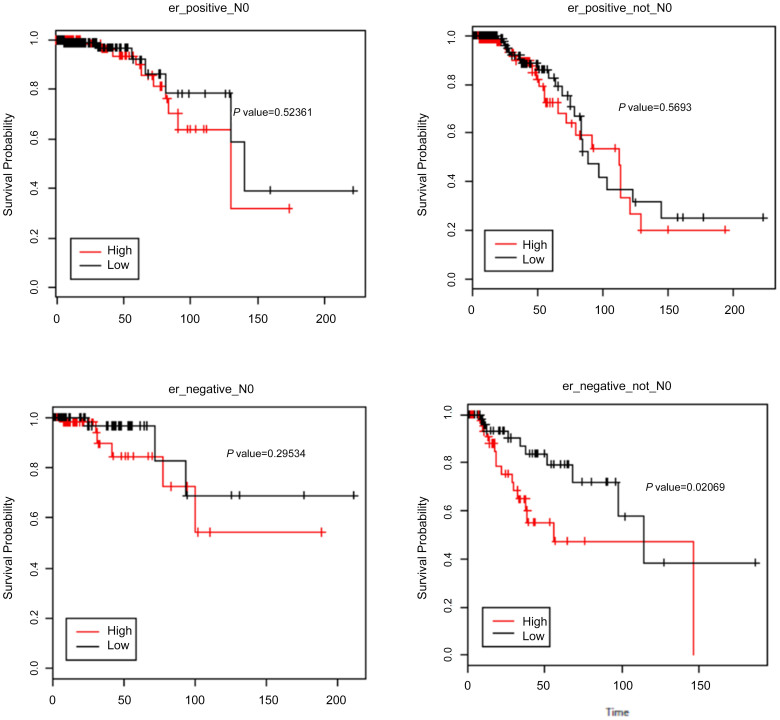
Kaplan–Meier overall survival in regards to *HOTAIR* expression and nodal status. Expression above median is not associated with poor outcome in ER-positive (regardless of lymph node status) (A & B). In ER-negative cases (C & D), high *HOTAIR* was only associated with poor outcome in lymph node–positive tumors.

We further assessed the correlation of *HOTAIR* expression with overall survival and lymph node status using TCGA dataset. High expression of *HOTAIR* was not associated with a greater likelihood of nodal metastases. Further subset analysis of the nodal involvement showed significance only in extensive node positivity (N3) when compared with N0 (*P* = 0.0049) and N1 (*P* = 0.00035) (Figure 3). In TCGA dataset, *HOTAIR* was not associated with prognosis in ER-positive patients (node positive and negative). High levels were found to be associated with worse prognosis in ER-negative/ node–positive patients (*P* = 0.02) but not in node-negative patients (*P* = 0.2) (Figure 4A–D).

## Discussion

Metastasis is the leading cause of breast cancer mortality. The prevention and treatment of metastasis, however, remains a significant clinical challenge. The spread of breast tumors to local and regional lymph nodes is an important means of tumor dissemination. The presence and the number of involved lymph nodes remains the single best indicator of whether or not the cancer has become widely metastatic. Identification of the underlying molecular mechanisms of lymph node metastasis and a better understanding how to modulate these will be a significant step in the goal of prevention of metastases.

Recent studies have shown that lincRNA is a novel class of molecules that regulates cancer progression and metastasis^27^. LincRNAs can serve as scaffolds to control chromatin states and epigenetic changes^17^,^20^. LincRNA *HOTAIR* was shown to regulate metastatic progression by reprogramming the chromatin state^17^,^20^. The high expression of *HOTAIR* was a significant predictor with poor prognosis and metastasis in breast carcinomas^17^. Further analysis performed by the same group showed that *HOTAIR* was also increased in the metastatic carcinomas when compared in matched primary and metastatic cancers^26^. A separate study found *HOTAIR* expression to be an important independent indicator for predicting metastasis, especially in ER-positive breast cancer patients^28^.

The current study investigated the role of *HOTAIR* expression in relation to nodal metastases in a breast cancer TMA cohort. Using RNA-ISH, in situ overexpression of *HOTAIR* was not associated with nodal metastases. We also did not observe any association with age, tumor grade, ER, and HER2. The difference in observed results could be due to a number of parameters including assay methods and size of cohort including the number of ER-positive cases. In the original study, Gupta et al identified increased *HOTAIR* expression in primary and metastatic breast cancers using RT-PCR based methods^17^. The same group (Chisholm et al) developed an in situ hybridization assay using well-established cell line controls^26^. In this followup study, they observed a trend of higher *HOTAIR* expression in the metastatic than in the primary breast cancers. Importantly, they could not confirm the survival data reported in the original study. However, based on the analysis of only 6 patients of the original 243 cases, they reported a trend for tumors overexpressing *EZH2* and *HOTAIR* to have a poor prognosis. Sorensen et al analyzed the expression of *HOTAIR* in a case control design study using an Agilent-based microarray platform^28^. They found high *HOTAIR* expression in primary tumors from patients who developed metastases as opposed to patients who did not. The association was significantly in patients with ER-positive tumors, but not in ER-negative tumors^28^. Lu et al analyzed the expression of *HOTAIR* in a series of 348 patients using quantitative RT-PCR^22^. They did not find a significant association of *HOTAIR* expression with prognosis in univariate analysis. These studies together indicate that the differences observed cannot be entirely explained by assay method variability.

The size of the cohort and the distribution of cases based on molecular classification and nodal positivity could explain some of the observed differences. To circumvent these issues, we analyzed the expression of *HOTAIR* in TCGA dataset (n = 952) and correlated it with nodal metastases and overall survival. Consistent with our in situ hybridization data, high *HOTAIR* expression in the entire dataset was not associated with nodal involvement (*P* = 0.33). High *HOTAIR* was associated with worse outcome in patients with ER-negative breast tumors (*P* = 0.018), but not in ER-positive patients (*P* = 0.41). This observation is in contrast to the results reported by Sorensen et al but are similar to those reported by Lu et al^28^,^29^. Some of the differences could be explained by the size of the cohorts, relative proportions of ER-positive and ER-negative patients, and the endpoints used. The endpoint in the current study is overall survival while many of the studies used distant metastases-free survival.

Association of high *HOTAIR* expression was observed with poor overall survival in ER-negative tumors; interestingly, this was observed only with patients having nodal metastases. It has been well documented that nodal involvement in triple-negative tumors is less prognostic in ER-negative tumors as opposed to ER-positive tumors. Several studies have reported that triple-negative cancers have less nodal metastasis, although the tumors are categorized as high grade^30^,^31^,^32^,^33^.

It is thus possible that the high expression of *HOTAIR* in ER-negative tumors could be an indicator of activation of pathways associated with lymphatic metastases rather than vascular metastases.

A strong association was seen between *HOTAIR* expression and histology. Ductal carcinomas (not otherwise specified) were less likely to have high levels of *HOTAIR* compared to special histological types such as lobular carcinomas (*P* < 0.00001). This finding is somewhat in variance with the high expression seen in ER-negative tumors.

In summary, our study shows that the prognostic role of *HOTAIR* expression is more or less restricted to ER-negative, lymph node–positive tumors, where its expression could possibly be used as a potential prognostic marker identifying patients at greater risk for poor overall survival. It does not seem to be useful in prognostication of ER-positive breast cancer or identification of patients likely to have nodal metastases. Given the well-recognized followup limitations of TCGA cohort, further studies are necessary to clarify the role of *HOTAIR* in metastases in cohorts well annotated for tumor histology, nodal status, and survival information.

## Methods

### Patient Cohort and Tissue Microarray (TMA)

Appropriate Institutional Review Board (IRB) approval from the Indiana University Research Ethics Committee was obtained. The methods were carried out in "accordance" with the approved IRB guidelines; informed consent was obtained from all subjects. A TMA was constructed from 133 consecutive patients with breast cancer. The procedure involved extracting 1 mm punches from tumors and implanting them in a new paraffin block. The array information was recorded in an Excel sheet. The cohort consists of 133 women with diagnosed with invasive carcinoma in the same year. The TMA was constructed using duplicate 1 mm cores from tumors. Clinical data with respect to age, tumor size, and ER and HER2 expression status were collected from the clinical charts. The ER and HER2 analyses were performed in a CLIA-certified lab using cutoffs recommended by the current ASCO-CAP guidelines^34^,^35^. All patients had undergone some form of nodal assessment (either sentinel node biopsy or axillary nodal sampling); none had received neoadjuvant chemotherapy.

### RNA In Situ Hybridization Assay (RNA ISH)

In situ detection for *HOTAIR* was performed using the RNAscope (Brown) FFPE kit (Advanced Cell Diagnostics, Hayward, CA) according to the manufacturer's instructions. Briefly, TMAs were cut in 4 μm thick sections. The tissue was baked for 1 hour at 60°C. The samples were then placed in Cytosol and brought down to water. Solution 1 was applied for 15 minutes at room temperature. The tissue sections were boiled at 95°C for 15 minutes in solution 2. Solution 3 (protease treatment) was then applied at 40°C. The provided probe and probe solution were applied. The slides were covered with agarose gel and placed in a rack at 40°C for hybridization for 2 hours. The tissue samples were then washed in the provided washing buffer. The 3,3′-diaminobenzidine solutions A and B were mixed in equal volume and left on for 10 minutes at room temperature. Hematoxylin counterstaining was performed.

A provided positive control probe ubiquitin C was used. For negative control, the enclosed negative control probe was applied. In addition, samples where the probe was omitted were included to exclude background staining.

The slides were independently evaluated by two separate observers (ITV and SB). Positive staining was indicated by signals as brown punctate dots present in the nucleus and/or cytoplasm as described in prior studies^26^. The number of signal staining was counted in 100 tumor cells. The study was performed on tissue microarrays (TMAs) which had 1 mm cores. This relatively small tissue core in many ways circumvented issues related to hotspots and tissue heterogeneity. The number 100 was chosen to ensure good representation of the tumor without loss of “huge” number of cases. The continuous number was categorized into four categories for statistical analysis. These categories were 0 = less than 1 signal per 100 cells; 1 = 1–10 signals; 2 = 11–100 signals, and 3 = > 101 signals in 100 cells.

The expression of the *HOTAIR* lincRNA was primarily correlated with nodal status. The secondary endpoints included correlations with other clinicopathological parameters such as age, tumor size, grade, and ER, PR, and HER2 expression status.

### Analysis of The Cancer Genome Atlas (TCGA)

To validate the clinical relevance of *HOTAIR* levels in a larger cohort of breast cancer, we obtained the normalized levels of *HOTAIR* expression (Level 3 data) in 952 breast cancer patients enrolled in TCGA database breast invasive carcinoma study (available at https://tcga-data.nci.nih.gov/tcga/tcgaHome2.jsp). Patients with breast cancer were categorized based on the ER status (n = 924; 656 ER positive), HER2 (n = 336; 145 HER2 positive), and lymph node status. The clinical information for each patient was also obtained. To model survival, gene expression at or below median was considered low and above median was considered high. Overall survival was calculated from the date of initial diagnosis of breast cancer to disease-specific deaths (patients whose vital status is termed dead) and months to last followup (patients who are alive). Kaplan–Meier survival analysis was used to estimate association of *HOTAIR* expression with survival of patients and with ER, HER2, and nodal status. The “survival” package in R (R Foundation for Statistical Computing) was used for statistical analyses.

### Statistical Analysis

Data were analyzed using Statistical Package for Social Sciences v.17.0 (SPSS Institute, Chicago, IL, USA) software to determine the correlations between *HOTAIR* and multiple clinicopathological parameters. Fisher exact test and chi-squared test were performed. All *P* values were two-sided, and 0.05 was taken as the significance level. Survival analyses by Kaplan–Meier method were performed for both epithelial and stromal cells as appropriate. The differences in survival were tested using the Log rank test.

## Author Contributions

Y.G.-P. and S.B. designed, interpreted the analyses, and wrote the main manuscript. I.T.V. and S.B. evaluated the RNA-ISH TMA cohort data, Y.N. and S.C.J. did the TCGA analysis. All authors reviewed the manuscript.

## Figures and Tables

**Table 1:** Clinicopathological characteristics of the 94 cases that were analyzable for the expression of *HOTAIR*

	Negative (0 or 1)	Positive (2–3)	*P* value
Premenopausal	16	15	1
Postmenopausal	31	32	
Tumor size ≤ 2 cm	27	34	0.194
Tumor size > 2 cm	20	13	
Grade 1	12	14	0.902
Grade 2	19	17	
Grade 3	16	16	
IDC	37	41	0.557
ILC	5	3	
Others	5	3	
Node-negative	28	34	0.276
Node-positive	19	13	
ER-negative	10	13	0.632
ER-positive	37	34	
HER2-negative	40	7	1
HER2-positive	39	8	

*IDC, invasive ductal carcinoma; ILC, invasive lobular carcinoma.*
